# Supplementary material for: Item-level psychometrics of the Ascertain Dementia Eight-Item Informant Questionnaire
Source: PLoS One. 2022 Jul 5;17(7):e0270204. doi: 10.1371/journal.pone.0270204 (PMC9255723; doi:10.1371/journal.pone.0270204)
Supplement: S1 Table — (DOCX) [file pone.0270204.s001.docx]

**S1 Table. Results of the confirmatory factor analysis of two estimation methods**

| Estimation Method | $x^{2}$statistics | Degree of Freedom | *p*-value | CFI | TLI | RMSEA (90% CI) |
| --- | --- | --- | --- | --- | --- | --- |
| WLSMV | 41.015 | 20 | 0.0037 | 0.995 | 0.993 | 0.051 (0.028, 0.073) |
| WLS | 31.459 | 20 | 0.0483 | 0.995 | 0.993 | 0.038 (0.003, 0.062) |

WLS, weighted least squares; WLSMV, robust weighted least squares
