# Supplementary material for: Item-level psychometrics of the Ascertain Dementia Eight-Item Informant Questionnaire
Source: PLoS One. 2022 Jul 5;17(7):e0270204. doi: 10.1371/journal.pone.0270204 (PMC9255723; doi:10.1371/journal.pone.0270204)
Supplement: Attachment — Submitted filename: feedback to authors.docx [file pone.0270204.s003.docx]

**Title of the manuscript**

Item-level Psychometrics of the Ascertain Dementia Eight-Item Informant Questionnaire

**All over comments/suggestions**

The item-level psychometrics of the Ascertain Dementia Eight-Item Informant Questionnaire (AD-8) will be a great asset for the scientific community to use in clinical and non-clinical areas. It was an interesting tool you select and thank you.

Dear Author, I am not such an expert on such type of tool analysis procedure. But I will try my best to maximize the outcome.

It was better to tell as to why you selected this tool instead of others in your literature part and discussion section.

Additionally, it was more strong evidence if you incorporate additional data (Not only 2019 Data Source).

**Major concern**

- Dear author, I just put my minor comments in the main document you can get it. But as for me, I didn’t get any clue why you choose this one?
- Because I didn’t get access to the full document I failed to see all over model fitness. So could you attach the summary table on your main manuscript and attach to me the raw data to see your model fitness test?
- Dear Author, if possible, could you please add your structural equation model to the manuscript?

If I may not mistake, Rasch analysis was used for categorical data, But the AD-8 rating scale is ordinal data? Could you please clarify this one?

With best regards!!

Mohammed Hassen (Assistant professor, Ph.D. Candidate).
