# Supplementary material for: Item-level psychometrics of the Ascertain Dementia Eight-Item Informant Questionnaire
Source: PLoS One. 2022 Jul 5;17(7):e0270204. doi: 10.1371/journal.pone.0270204 (PMC9255723; doi:10.1371/journal.pone.0270204)
Supplement: Attachment — Submitted filename: responses to plos one.docx [file pone.0270204.s005.docx]

Dear the author

Thank you for your clear and scientific manuscript. Much appreciated your effort on adding value for the scientific community. Totally agreed on your response and revision.

I am just to say congratulation for the paper submitted to your journal entitled “ Item-level Psychometrics of the Ascertain Dementia Eight-Item Informant Questionnaire” .

I am happy for the correction did on sampling and attached the structural equation model.

Have a good day.

Mohammed Hassen Salih (Assistant professor, Ph.D. Fellow)
